# Perception of Nursing Staff in ICU Regarding Measures to Prevent Hospital-Acquired Infections: A Qualitative Approach

**DOI:** 10.7759/cureus.33029

**Published:** 2022-12-28

**Authors:** Ioanna Dimitriadou, Sarantis Pittas, Alexandros Sidiropoulos, Olga Zarkali

**Affiliations:** 1 Nursing, Health Center of Veroia, Veroia, GRC; 2 Nursing, University of Thessaly, Larisa, GRC; 3 Cardiology, General Hospital of Veroia, Veroia, GRC

**Keywords:** intensive care units, nursing staff, cross-infection, infection prevention and control, hospital acquired infections

## Abstract

Background

Hospital-acquired infections are a common problem in Intensive Care Units and are associated with significant morbidity, mortality, and cost of hospitalization. Despite the development of prevention and control strategies, the incidence of hospital-acquired infections remains consistently high in ICUs and is often associated with the practices of healthcare professionals in patient care.

Purpose

The aim of the study was to investigate the perceptions of ICU nursing staff regarding measures for the prevention of hospital-acquired infections.

Methods and materials

This is an ethnographic qualitative study. It was performed in the Intensive Care Unit and the Cardiac Care Unit of a General Hospital in North Greece. Seventeen nurses recounted their perceptions regarding infection prevention and control through semi-structured interviews. Thematic analysis using inductive and deductive approaches was conducted. This manuscript adheres to the Consolidated Criteria for Reporting Qualitative Research (COREQ) guidelines.

Findings

The two basic themes that emerged are (1) infection prevention measures in practice, and (2) factors that affect compliance with infection prevention measures.

Conclusions

The results of this study demonstrate the need for training and compliance of ICU nursing staff regarding measures to prevent hospital-acquired infections. There are several barriers that need to be addressed, such as culture, working conditions, and communication problems through documented interventions in the education, working environment, and professional identity of nursing staff.

## Introduction

Hospital-acquired infections (HAIs) are a global health challenge because they affect the health and safety of both patients and hospital workers [1]. They are the primary cause of the increase in morbidity and mortality of patients. And their treatment results in high cost, increased need for advanced medical care, and use of expensive antibiotics, which may lead to the emergence of other antimicrobial-resistant organisms [2]. HAIs are particularly pervasive during acute critical care, where patients are prone to infection due to the host’s reduced defence mechanisms caused by the severity of illness, underlying diseases (e.g., diabetes and cancer), the presence of multiple invasive devices resulting in disruption of anatomical and immunological protective barriers, and the administration of various drugs [3].

According to the European Centre for Disease Prevention and Control (ECDC), HAIs in Greece are a major healthcare challenge, as approximately one in ten hospitalized patients (about 9%) develop an HAI. In hospitals treating critically ill patients, the rates increase dramatically, with a rate of 50% in adult ICUs and 30% in neonatal ICUs [4].

The ICU is the ideal environment for the cross-transmission of multi-resistant pathogens. The main mode of spread by cross-transmission is the colonization of the hands of healthcare workers (HCWs) and transfer from patient to patient either through the hands or gloves, especially when the gloves are not replaced after use [5]. For this reason, infection prevention and control (IPC) strategies and universal implementation of control programs become a top priority. However, these changes have not resolved the gap between the evidence and clinical practice, particularly in terms of healthcare workers' (HCW) behavioural change [6,7].

The purpose of this study was to investigate the perception of nursing staff in ICUs regarding measures to prevent HAIs. We also intended to explore the facilitators and barriers to nurses’ adherence to evidence-based clinical practice in preventing HAIs in a Greek hospital. The use of qualitative research allowed us to identify behavioural patterns and values about IPC policies and practices.

## Materials and methods

In this qualitative study, we used ethnographic data-collection techniques, including semistructured individual interviews with open-ended questions. We adhered to the Consolidated Criteria for Reporting Qualitative Research (COREQ) guidelines [8].

We purposively sampled nurses from the Intensive Care Unit (ICU) and Cardiac Care Unit of a general hospital in northern Greece. We selected our sample to include nurses of both genders working in the ICU and able to cooperate in the interview. After selecting the sample, we asked them whether they agreed to participate. Also, we explained the purpose of this study. During autumn 2022, we approached thirty-three nurses, of whom only seventeen agreed to participate. Four out of the seventeen were men and thirteen were women aged twenty-six to fifty-nine. All nursing staff working on this ward were on a rotating roster during the data collection. The interviews were conducted by two interviewers using an interview guide (Table 1).

**Table 1:** Guide for the semi-structured interview

1. How would you describe your typical workday?
2. What is your training on the prevention of hospital-acquired infection?
3. Do you know any standards or guidelines related to infection prevention and control?
4. What infection prevention measures and practices do you apply in daily practice?
5. How do you evaluate the infection prevention measures you follow?
6. Do you think hospital-acquired infections are a problem in your hospital? Can you explain why?
7. What infection prevention measures and practices your colleagues use?
8. How do you evaluate the infection prevention measures followed by your colleagues?
9. What factors do you consider to be related to compliance or non-compliance with these measures? Can you think of specific examples?
10. How do you think infection prevention and control measures could be better implemented?
11. Is there anything else about preventing hospital-acquired infections that you would like to share with me?

The interview guide contained questions in several areas, including education about infection-prevention measures, implementation, and evaluation of those measures, impact of hospital-acquired infections on the work environment, implementation and evaluation of infection-prevention measures among colleagues, factors influencing the implementation of those measures, and ways to improve the implementation of the measures. The interviews were of thirty to forty-five minutes duration. The discussions were not recorded because the participants reported that they felt comfortable, and therefore we kept extensive notes during the interviews. The interviews were recorded and transcribed verbatim.

We provided the informants with written and oral information about the study, including their right to withdraw and their confidentiality and anonymity. They also signed an informed consent form.

Data collection, interview transcriptions, and analysis occurred concurrently to monitor the progress of themes emerging from individual interviews. We aggregated data and coded the data thematically using thematic analysis, which is the most common method of data analysis in qualitative research and aims to analyse and systematically record the coding and themes that emerge from data collection, namely, personal interviews, focus groups, and observations. We considered this method the most suitable for the analysis of the written discourse resulting from the nurses' interviews to clarify their perceptions regarding the measures to prevent hospital-acquired infections.

The analysis began with transcribing the recorded interviews, reading the written text, and writing short notes summarizing the text. Then we collected the initial codings (i.e., the words and phrases recorded by the researcher) and recorded them in a self-contained text with duplicate entries removed. Next, we grouped the initial codings into broader categories, based on similarities and differences in the content, in that way forming the themes based on the research axes of the studies, which are inextricably linked both to the theoretical background of the research question and to the personal judgment of the researcher.

## Results

We classified our findings into two main themes and four categories, highlighting and recording the perceptions of the participants regarding the measures to prevent hospital-acquired infections. The categories emerged based on the questions raised as summarized in Table 2.

**Table 2:** Themes, categories, and link with the research questions HAI: hospital-acquired infection

Themes	Categories	Link with the research questions
HAIs prevention measures in practice	Education and knowledge regarding HAIs and their prevention measures.	1. What is the training of nurses regarding measures to prevent HAIs?
	Preventive measures of HAIs in daily practice	2. What measures related to the prevention of HAIs do nurses use?
Factors influencing the implementation or non-implementation of infection prevention measures	Factors influencing the implementation or non-implementation of infection prevention measures	3. What are the factors related to the implementation or non-implementation of these measures?
	2. Strategies to improve the implementation of infection prevention measures	4. With what practices can the implementation of infection prevention measures be improved?

HAI prevention measures in practice

The first theme describes participants’ infection prevention and control measures related to key recommendations in current evidence-based clinical practice guidelines. There were two categories: education and knowledge regarding HAIs and their prevention and HAI prevention measures in daily practice.

Education and Knowledge Regarding HAIs and Their Prevention

Education and knowledge play an integral and primary role in nursing-staff compliance with guidelines. From the collected responses by the population under investigation, we found that the training of nursing staff comes mainly from theoretical training and skill development during their studies, while some respondents also referred to the help of colleagues during their first days of work.

The training I have received is mainly from my school. When I started working, I had a briefing from the infection control nurse, without practical and I started working in the ICU. In the ICU area, there are procedure documents. I find it easier to ask someone. 

During the interviews, most participants emphasized the absence of continuing education after their schooling, both on a theoretical and practical level. All participants considered their school education to have been adequate. However, most referred only to hand hygiene and the use of gloves. Few reported learning about the infections that thrive in the ICU and the interventions to prevent them, while none reported learning about the colonization and spread of multidrug-resistant pathogens. Only one mentioned the ICU environment and the use of external devices or the need to adopt infection-prevention measures in the patients’ environment. 

I remember several things from my school, and especially to wash my hands and change gloves from patient to patient. 

Preventive Measures of HAIs in Daily Practice

In this category, we detail the infection-prevention measures used by participants in daily practice. More generally, most participants referred to hand hygiene and changing gloves from patient to patient, while some referred to central venous catheters and ventilator care.

I always use hand hygiene. I never touch the patient with bare hands and make sure to change gloves when I am going to another patient.

I use hand hygiene according to the five moments of hand hygiene. I use gloves during my contact with the patient and his environment. I use sterile gloves, protective equipment and surgical field in every operation that requires aseptic technique. I place clear pads on central venous catheters and arterial lines to assess the entry site without contaminating the area.

Most participants reported that they practice hand hygiene, but some did not specify when they do this. In addition, while most reported using gloves, few mentioned using other personal protective equipment such as medical aprons. Furthermore, although the patients who were hospitalized in the ICU were critically ill and had several interventional devices, few reported using aseptic techniques during the placement and care of interventional devices. Finally, none mentioned the cleaning of life support devices or the patients’ environment.

Evaluation of Infection Prevention Measures

Participants answered questions related to the evaluation of measures they use in daily practice. Most stated that the infection prevention measures they used daily were adequate, were in line with guidelines, and protected patients and themselves from infection transmission. Typical statements included the following:

I consider the infection prevention measures I use to be adequate. Besides, I have been working in the ICU for several years, never had a problem with patients, nor myself.

I believe that the prevention measures I use are what they should be. That’s what I was shown and that’s what we all do here.

Interestingly, most respondents to this question were embarrassed. When they were asked to rate the infection and prevention measures they used in daily practice, they answered vaguely and briefly. All reported that the prevention measures they used were adequate and protected both patients and themselves. None expressed any doubts or deficits about the measures they used.

Infection-Prevention Measures Used by Colleagues

This subsection focuses on reporting on the infection prevention measures used by their colleagues. Most participants claimed that their colleagues had adopted in a general context the measures they used themselves. Below are some of their responses:

All colleagues use gloves during patient contact and practice hand hygiene. I have never seen anyone touching a patient without gloves. 

However, some participants had a different opinion:

Several people wear gloves when they will come in contact with the patient and then do not change them. Several times, when inserting central venous catheters, they do not use any personal protective equipment, do not use a surgical field, and generally do not do the insertion under aseptic conditions.

This question seems to have divided the participants and caused ambivalent feelings. Two completely different views emerged about the measures used in daily practice by their colleagues. Several respondents rationalized the behaviour of their colleagues, stating that they adhered to all infection prevention measures and that all nursing staff were compliant with the guidelines and protocols for the prevention of nosocomial infections in ICUs. On the contrary, some expressed a different opinion, referring to specific examples and situations where the necessary measures were not observed, and they generalized this behaviour as characterizing many of their colleagues in daily practice.

Evaluation of Infection Prevention Measures Among Colleagues

Finally, the last subsection concerns the evaluation of infection-prevention measures among colleagues. As in the preceding subsection, the answers diverged among respondents. Several evaluated positively the prevention measures used by their colleagues. Some of the most typical responses were as follows:

All nursing staff use infection prevention measures during patient care. All protocols related to hand washing, use and removal of protective equipment, adherence to aseptic technique during invasive procedures are followed. There is cooperation and continuous communication for any additional instruction.

 In contrast, some participants expressed completely different views. Some of their responses were as follows:

Unfortunately, several colleagues circumvent infection prevention measures and guidelines on preventing spread of microorganisms. There are workers who do not use gloves when in contact with the patient and his/her environment. Even, some do not change equipment when they finish caring for one patient and move on to the next. I believe, these are examples of basic infection prevention strategies in hospitals.

Factors influencing compliance or non-adherence to infection prevention measures

The second theme that emerged from the research questions and the responses of the respondents concerned the factors associated with adherence or non-adherence to infection-prevention measures. In general, the main factors mentioned by participants were knowledge, on-the-job training, culture, lack of staff, workload, and poor cooperation with other disciplines. Remarkably, spontaneously all participants mentioned factors related to non-adherence to prevention measures. None of the participants mentioned any factors that positively influenced adherence to prevention measures in daily practice. Below are some typical descriptions of the participants:

Unfortunately, no further training is promoted by our employer, the shifts and the rotating hours exhaust us and the financial cost for some of us is prohibitive. Therefore, I believe our knowledge is incomplete, which is reflected in our adherence to infection prevention measures.

I believe that infection prevention measures are not adhered to because staff were not trained by the employing organization. When I started working, I was brought to the ICU without being trained or talked to about nosocomial infections. The department told me I would learn while working.

Ways to Improve the Implementation of Infection-Prevention Measures

Regarding ways to improve the implementation of infection prevention measures in the hospital setting, there were extensive responses from participants. Most focused on continuing education, rewards, better working conditions, and strengthening the role of the nurse. Some of their statements are as follows:

Well, in order to improve the implementation of infection prevention measures the primary objective is continuing education. To have the opportunity to be trained both at the cognitive level, to participate in courses, briefings, conferences, to be continuously updated by the Education Office and the Hospital Acquired Infections Committee and to be trained in clinical skills. This project should have a continuity and, in my opinion, should be interactive. I think, it would greatly contribute to improving the implementation of infection prevention measures.

Rewards in the workplace would be a key incentive for the implementation of infection prevention measures. Reward could be related to job progression, responsibility, trust in the employee. Following on from the reward, the general strengthening of the nurse’s role in the process. It is therefore necessary to strengthen their role in the prevention of infections in the hospital setting in order to look for the real problems, priorities improvement strategies and implement them in daily practice.

Nursing staff seem to know in what ways the implementation of infection prevention measures can be improved. In general, participants made realistic suggestions for improving those measures in the hospital environment. Characteristically, none of the participants mentioned improving their monetary remuneration, while all of them mentioned improving working conditions.

## Discussion

In Greece about 10% of hospitalized patients will develop an in-hospital infection, and the association with pathogenic multidrug-resistant microorganisms increases the rate of hospitalization days, costs, morbidity, and mortality. Well-executed infection prevention and control practices, such as hand hygiene, surface cleaning, proper use of personal protective equipment, and isolation of patients with contagious infections are some of the most successful and cost-effective ways to solve the chain of transmission in the hospital setting [4]. The importance of adhering to infection prevention and control guidelines is widely recognized, yet their adoption is still suboptimal among healthcare professionals [9]. Recently, in a qualitative survey, an attempt was made to investigate the perceptions of health professionals on prevention measures in neonatal intensive care units in Greece.** **However, the views of nurses working in neonatal ICUs (NICUs) regarding infection prevention measures have not been investigated [10].

Measures to prevent nosocomial infections in practice

Nursing staff are directly involved in patient care in the hospital. Thus they play an important role in the transmission of nosocomial infections, and their compliance with infection control measures is essential to prevent the spread. Education and knowledge perform an integral and primary role in nursing staff compliance with guidelines. Studies have revealed that nursing staff have a knowledge deficit regarding aseptic technique maintenance, and risk factors and guidelines [11,12]. The key to avoiding infection in nurses lies in hand hygiene. Other recommended practices such as antimicrobial stewardship and bundle measures did not receive consistent focus. Moreover, it has been observed that even for nursing staff working in critical posts, such as infection control nurses and ICU nurses, there is no continual training in the theoretical and practical application of infection prevention measures [13].

The results of our study are consistent with the existing literature. Nurses seem to lack knowledge about infection prevention measures and focus mainly on hand hygiene, neglecting other important parameters and policies to reduce spread in ICUs. In addition, the education of nursing staff is mainly offered in nursing school, and the absence of training by their employer is evident. However, it is surprising that while there are policies and guidelines that are in line with modern standards, nurses conspicuously ignore them.

Specifically, in ICUs, there are guidelines covering a wide range of topics related to the spread of pathogens, patient and employee safety, and infection prevention and control measures, yet they are kept in a document repository. Staff, especially new employees, use experienced colleagues as a source of knowledge but do not seek out the guidelines. In a study investigating the facilitators and barriers to adherence to infection prevention guidelines in the surgical field, nursing staff looked to other colleagues for action and did not use the procedural protocols [11,14]. This demonstrates the existence of a ‘culture’ among nursing staff of seeking knowledge among colleagues with several years of experience, and of not seeking such knowledge from either the applicable guidelines.

Although knowledge is considered essential for optimal healthcare delivery, knowledge alone is not sufficient. The ability to apply this knowledge is essential to ensure that the highest standards of clinical practice are applied, particularly when caring for the critically ill. An observational study investigating the practical application of infection prevention measures by nursing staff found that there were several discrepancies among basic practices such as hand hygiene, aseptic techniques, and the use of simple and sterile gloves [15].

According to the results of our study, nurses refocused on hand hygiene and glove use, ignoring other infection prevention and control policies. However, there was ambiguity about when they performed hand hygiene and in what way, when they used simple gloves and sterile gloves, and when and how they disposed of them. According to Lin et al. [11] adherence to hand hygiene and glove use can be influenced by a multitude of factors, such as environmental factors (availability in facilities, hand-washing products, and reminders in key workplaces), organizational factors (leadership in promoting infection prevention measures), and educational factors. These factors could partly explain why nursing staff focus on specific procedures.

According to the study by Jackson et al. in the UK designed to investigate nurses’ infection prevention behaviours, nurses could not discern similarities in their practices to those of colleagues. They identified incorrect practices of their colleagues related to the use of personal protective equipment and rated this practice as ‘poor’ and as a means of increasing the risk of infection. It is particularly interesting that when they took similar actions they understood and justified their own behaviour. This justification rationalizes their own practices and behaviours [16].

The results of our study are consistent with the literature, as nurses positively evaluated the infection prevention measures that they used in daily practice without reporting any deficits or interventions. However, it is interesting that the participants gave quite short answers and descriptions, and there was an atmosphere of embarrassment. Furthermore, some of the nurses accurately identified incorrect practices used by their colleagues in their daily routine and evaluated these behaviours negatively because they had an impact on inpatients, their workplace, and the employees themselves. But some of the participants positively evaluated the prevention measures used by their colleagues without any reference to any risky behaviour, rationalizing, in the same way, the practice of their colleagues.

These results demonstrate similarities with the study by Morrow et al. who examined the causes of behaviour in relation to methicillin-resistant *Staphylococcus aureus *(MRSA) control. Health professionals attributed MRSA acquisition to the actions of others but showed a favourable bias towards their own practices [17].

Factors influencing compliance or non-compliance with infection prevention measures

Factors influencing compliance or non-adherence to infection-prevention measures seem to play an important role in the behaviour of nursing staff in ICUs. The results highlighted a significant number of barriers that included staff knowledge and training, culture, lack of staff, and workload. Nurses extensively referred to improving the practice of infection prevention and to their function as facilitators [13].

The results of our study highlight how important education and knowledge are for nursing staff, as knowledge was identified as a barrier by all participants. They emphasized the need to create continuous education both theoretical and practical, feedback from the team and infection-control nurses, and financial support from the provider to seek training programs. Education and knowledge acquisition needs to be a systematic process with targeted interventions in clinical practice. Knowledge is an essential tool for understanding the spread, potential risks, and compliance with guidelines.

Culture was identified as a major barrier to guideline adoption. More generally, there is a belief that a nurse’s clinical experience can replace health poles, resulting in a deeply entrenched resistance to change. This finding agrees with that of Triantafillou et al. that culture influences compliance with guidelines and that there is a hierarchy that does not allow new employees to make recommendations to older workers or nursing staff to intervene with physicians regarding anti-dispersal measures [10].

According to researchers, systemic deficiencies in infection control and prevention in hospitals are related to a lack of staff, increased workload, and limited financial resources [12]. In our study, participants did not mention of lack of resources and financial support, which is surprising because the healthcare system in Greece has been tested and weakened in previous years. However, an important parameter that hinders the implementation of infection prevention measures is the lack of personnel and as a side effect the workload, due to which many procedures and applications are omitted. Nursing staff believe that better working conditions would be an important facilitator for better implementation of prevention measures in daily practice.

The literature highlights the need to strengthen the role of health professionals working in critical positions, such as infection control nurses and ICU nurses, through the implementation of strategies resulting from the assessment of patients and their potential risks [10,13]. These findings are consistent with our present study, as nurse empowerment, assuming responsibility, and taking initiative seem to play a crucial role in nurses’ professional lives. It is important to recognize nurses’ contributions to infection prevention and control and their critical role in ICUs.

Limitations

This qualitative study has several limitations. First, during sample collection, the number of available candidates who could participate in the study was limited. In addition, the face-to-face interview process was an unfamiliar process to the potential participants, resulting in several of them refusing to participate. As a side effect, the number of participants was less than compared to the total number of ICU nurses. Finally, the interviews were conducted during the SARS-COV-2 pandemic, resulting in ICU staff being trained at regular intervals on infection prevention measures and being vigilant about the transmission of infections in the ICU setting.

## Conclusions

The aim of this research was to investigate the perceptions of nursing staff in the ICU regarding infection prevention measures. The focus on ICU is due to the specific epidemiology and patient profile. The qualitative methodology and, more specifically, the 17 interviews conducted contributed to the spontaneous emergence of nursing staff perceptions around this much-discussed issue, which has been studied mainly through quantitative surveys based on epidemiological data. Thus, new aspects have emerged that allow us to deepen the knowledge, perceptions, and attitudes of nursing staff regarding infection prevention measures in the hospital setting, as well as evidence that confirms the findings of other studies in the relevant literature.

In conclusion, the results demonstrate the need for education and compliance of nursing staff with the measures for the prevention of nosocomial infections in ICUs. There are some barriers that need to be addressed, such as the culture of nursing staff in terms of compliance with infection prevention measures, working conditions, and poor communication between health professionals. Targeted interventions in education, improving the working environment, and strengthening the professional identity of nurses could contribute to compliance with prevention measures and the management of HAIs in ICUs.
